# The distribution characteristics of global *blaOXA*-carrying *Klebsiella pneumoniae*

**DOI:** 10.1186/s12879-023-08156-5

**Published:** 2023-03-29

**Authors:** Lingning Meng, Ziyao Liu, Chang Liu, Chuchu Li, Han Shen, Xiaoli Cao

**Affiliations:** 1 Department of Laboratory Medicine, Nanjing Drum Tower Hospital, the Affiliated Hospital of Nanjing University Medical School, Zhongshan Road 321, Gulou, Jiangsu Province Nanjing, People’s Republic of China; 2 The Precision Medicine Centre of Nanjing Drum Tower Hospital, the Affiliated Hospital of Nanjing University Medical School, Nanjing, Jiangsu China; 3 Department of Acute Infectious Disease Control and Prevention, Jiangsu, Provincial Center for Disease Control and Prevention, Nanjing, China

**Keywords:** *Klebsiella pneumoniae*, *OXA*, Sequence Type, Phylogeny, ST11

## Abstract

**Objective:**

To analyze the distribution of *blaOXA* among global *Klebsiella pneumoniae* and the characteristics of *blaOXA*-carrying *K. pneumoniae.*

**Materials and Methods:**

The genomes of global *K. pneumoniae* were downloaded from NCBI by Aspera software. After quality check, the distribution of *blaOXA* among the qualified genomes was investigated by annotation with the resistant determinant database. The phylogenetic tree was constructed for the *blaOXA* variants based on the single nucleotide polymorphism (SNP) to explore the evolutionary relationship between these variants. The MLST (multi-locus sequence type) website and blastn tools were utilized to determine the sequence types (STs) of these *blaOXA*-carrying strains. and sample resource, isolation country, date and host were extracted by perl program for analyzing the characteristics of these strains.

**Results:**

A total of 12,356 K*. pneumoniae* genomes were downloaded and 11,429 ones were qualified. Among them, 4386 strains were found to carry 5610 *blaOXA* variants which belonged to 27 varieties of *blaOXAs*, *blaOXA-1* (*n* = 2891, 51.5%) and *blaOXA-9* (*n* = 969, 17.3%) were the most prevalent *blaOXA* variants, followed by *blaOXA-48* (*n* = 800, 14.3%) and *blaOXA-232* (*n* = 480, 8.6%). The phylogenetic tree displayed 8 clades, three of them were composed of carbapenem-hydrolyzing oxacillinase (CHO). Totally, 300 distinct STs were identified among 4386 strains with ST11 (*n* = 477, 10.9%) being the most predominant one followed by ST258 (*n* = 410, 9.4%). Homo sapiens (2696/4386, 61.5%) was the main host for *blaOXA*-carrying *K. pneumoniae* isolates. The *blaOXA-9*-carrying *K. pneumoniae* strains were mostly found in the United States and *blaOXA-48*-carrying *K. pneumoniae* strains were mainly distributed in Europe and Asia.

**Conclusion:**

Among the global *K. pneumoniae,* numerous *blaOXA* variants were identified with *blaOXA-1*, *blaOXA-9*, *blaOXA-48* and *blaOXA-232* being the most prevalent ones, indicating that *blaOXA* rapidly evolved under the selective pressure of antimicrobial agents. ST11 and ST258 were the main clones for *blaOXA*-carrying *K. pneumoniae.*

**Supplementary Information:**

The online version contains supplementary material available at 10.1186/s12879-023-08156-5.

## Introduction

*Klebsiella pneumoniae* belonging to *Klebsiella spp.* is one of the important species of *Enterobacterale.* As one of the most common opportunistic pathogens, it is also a major cause of hospital associated infections, ranking in the top three causative agents in most hospital settings [1]. As we know that extended β-lactams and carbapenem are high-efficiency broad-spectrum antibiotics with high sensitivity to *Enterobacterale* and are commonly used to treat *K. pneumoniae* infections. Therefore, with the antimicrobial agents being extensively used in clinical therapy, extended-spectrum β-lactam (ESBL)-producing and carbapenem-resistant *K. pneumoniae* (CRKp) have rapidly developed and disseminated, leading to subsequently increased morbidity and mortality. Thus, they were taken as a critical public health threat by The World Health Organization (WHO) [2].

It has been well known that *blaCTX-M*, *blaTEM*, *blaSHV* and *blaOXA* were the most frequent ESBLs, *blaKPC*, *blaNDM* and *blaOXA-48* were the most common carbapenem-hydrolyzing β-lactamase (CHβLs) encoding genes widely prevalent in *K. pneumoniae* worldwide [3, 4]. In recent years, carbapenem-hydrolyzing oxacillinase, especially *blaOXA-48*-carrying *K. pneumoniae* has been increasingly reported worldwide, with Europe and Aisa being a hot spot for outbreaks of infection [5, 6], which endangers public health. However, information on the characterization of *blaOXA*-carrying *K. pneumoniae* is little. Unfortunately, the spread of *blaOXA-48* gene in Iran, as an endemic country, has not been investigated and only one study has been investigated. Iran, Span, Greece and China are countries with high prevalence of *blaOXA-48* gene [7]. So, in this study, we intended to know the changing epidemic characteristics and trends of *blaOXA* genes in *K. pneumoniae* which is helpful for the formulation of infection control measures.

Oxacillinases or OXA ß-lactamase was first recognized in the 1960s, displaying hydrolytic activity against penicillins and oxacillin. Afterwards, certain OXA ß-lactamases were found to inactivate the cephalosporins and carbapenems. To date, more than 940 types of OXA ß-lactamases have been found, showing heterogeneous substrate profiles. OXA ß-lactamases were divided into the 4 groups by Poirel and colleagues in 2010 [8]. Group I include OXA-1, OXA-2, and OXA-101 subgroups, belonging to acquired narrow-spectrum class D ß-lactamases without significant activity toward cephalosporins or the carbapenems. Group II is the acquired extended spectrum class D ß-lactamases hydrolyzing certain extended-spectrum cephalosporins (especially ceftriaxone and cefepime), which often originates from point mutations of narrow-spectrum class D ß-lactamases. However, structurally, certain extended-spectrum OXA enzymes are not related to the narrow-spectrum group I OXA-lactamases. Group III owns the ability to weakly hydrolyze carbapenems, but do not significantly hydrolyze the extend-spectrum cephalosporins. It is named carbapenem-hydrolyzing class D ß-lactamases and composed of 2 subgroups. One subgroup named the OXA-23-like enzymes, responsible for carbapenem resistance among *Acinetobacter spp*., the other subgroup is named the OXA-48-like enzymes, mainly found among the *Enterobacterale*s, especially in *K. pneumoniae*. Group IV forms parts of the chromosomes of various non-fermenting Gram-negative bacteria, which includes the OXA-51-like subgroup in the *Acinetobacter baumannii* complex.

Molecular studies displayed a frequent distribution of *blaOXA* including *blaOXA-1*, *blaOXA-2*, *blaOXA-48* etc. among *K. pneumoniae* [9]. Among the *blaOXA-48*-like CHβLs group, *blaOXA-48*, *blaOXA-181*, *blaOXA-232*, *blaOXA-204*, *blaOXA-162*, and *blaOXA-244* are the most common enzymes identified, and some of them showed an epidemic state, for example, it has been reported that *blaOXA-48* is endemic in Europe and Asia [5, 6]. *The blaOXA-162* and *blaOXA-244* (derivatives of *blaOXA-48*) were present in Europe [10]. The *blaOXA-181* and *blaOXA-232* were endemic in the Indian subcontinent and certain sub-Saharan African countries [11, 12]. Additionally, clonal dissemination of certain high-risk clones such as *K. pneumoniae* ST147, ST307, ST15, and ST14 play roles in the global dispersion of *blaOXA-48*-like CHβLs [12]. In recent years, detection of *blaOXA-48* and clonal dissemination of *blaOXA-48*-carrying carbapenem-resistant Enterobacteriaceae (CRE) has been reported in China [13]. However, the distribution of *blaOXA* among global *K. pneumoniae* is unknown and the information available is quite limited.

In this study, we firstly analyzed the distribution and phylogeny of *blaOXA* among global *K. pneumoniae* isolates. For the *blaOXA*-carrying strains, we further investigated the sequence type (ST) and the characteristics.

## Materials and Methods

### The acquisition and quality check of *K. pneumoniae* genomes

Aspera software was applied to download the *K. pneumoniae* genomes deposited in GenBank (https://www.ibm.com/aspera/connect/). A total of 12,356 complete genomes of *K. pneumoniae* strains were accessible. After the genome quality check by CheckM and Quest software, 11,429 qualified genomes were finally analyzed in this study.

### Standardized genome annotations

All the 11,429 genomes were annotated by prokaryotic genome rapid annotation software Prokka to analyze the *blaOXA* genotype, thus to avoid differences in genomic predictions with different annotation methods and to get the strains carrying *blaOXA*.

### Phylogenetic tree of *blaOXA* variants

Multiple sequence alignment for nucleotide sequences of the *blaOXA* gene was performed using Muscle [14], and then the GTRCAT replacement model was selected through the RaxML software [15]. In addition, 500 bootstrapping samples were set up to construct the preliminary phylogenetic tree, which was further imported into iTOL software to obtain the final one by setting bootstrap value being with less than 50 for the branches being deleted to obtain the final evolutionary tree [16].

### Analysis of sequence types (STs)

Seven housekeeping gene sequences (*gapA*, *infB*, *mdh*, *pgi*, *phoE*, *rpoB*, and *tonB*) and typing profile files of *K. pneumoniae* strains were obtained from MLST website (https://pubmlst.org). All the nucleotide sequences of 4386 *blaOXA*-carrying strains were analyzed using blastn tools in batches using housekeeping gene sequences as the database (parameters: value = 1e-5; identity = 100%; coverage = 100%).

### Investigation of antimicrobial resistance genes among *blaOXA*-carrying *K. pneumoniae*

The distribution of other resistance genes including CHβLs encoding genes, aminoglycoside and fluoroquinolone resistance genes was investigated by blastn tools. All the nucleotide sequences of *blaOXA*-carrying strains were analyzed with the sequence of antimicrobial resistant genes as database using blastn, and then the results were filtered (parameters: identity >  = 90%; coverage >  = 90%; value = 1e-5).

### Acquisition of strain meta information

Strain meta information such as isolation source, country, and date, etc. was extracted in batches from the downloaded gbk file by using perl script. Meanwhile, the corresponding cds, genes, contig quantity and genome length information of the genomes were also obtained from the prokka annotation file. All the information in addition to STs were integrated in an excel to facilitate the further analysis.

## Results

### OXA distribution among global *K. pneumoniae* strains

**Fig. 1:**
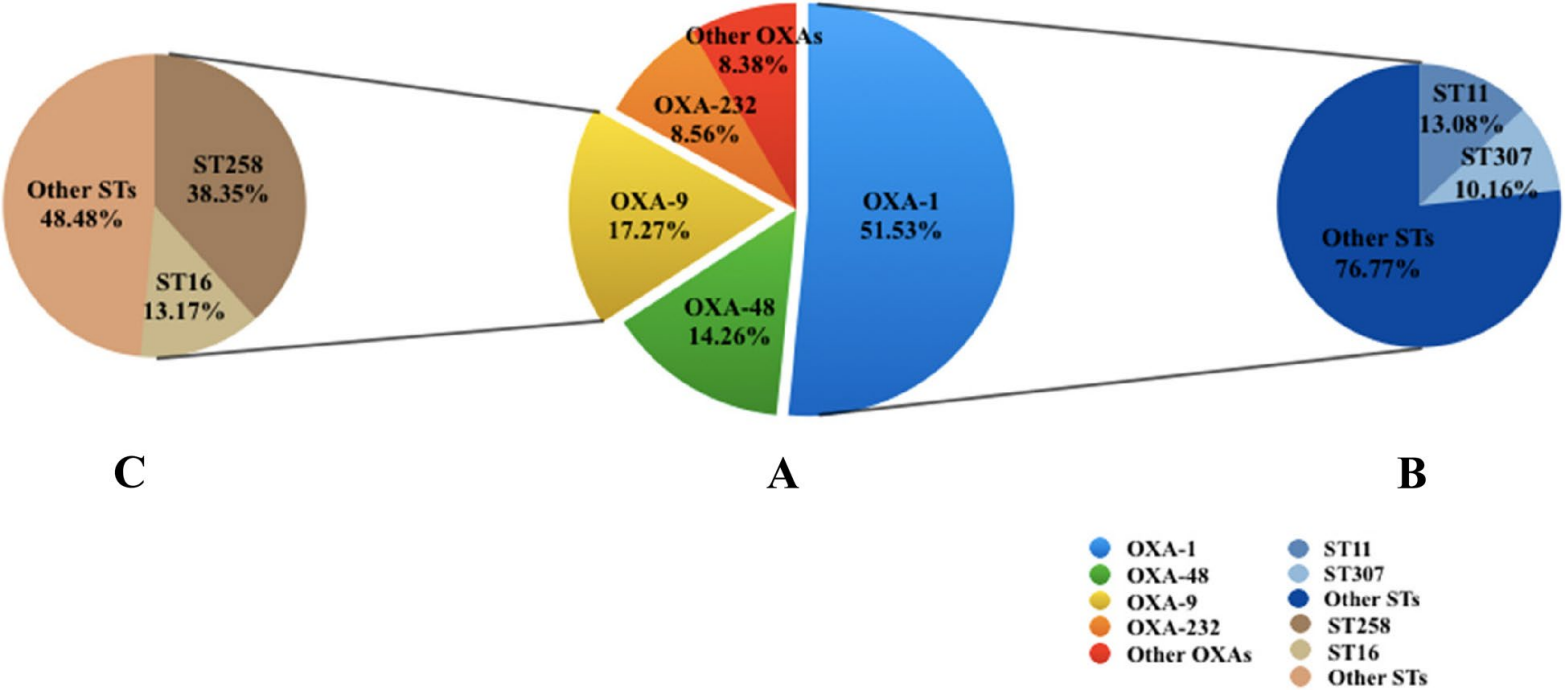
Distribution of *K. pneumoniae blaOXA* variants and sequence types

Among the 11,429 K*. pneumoniae* strains, 4386 ones carried a total of 5610 *blaOXA* variants which belonged to 27 genotypes of *blaOXAs*. The predominant was *blaOXA-1* (*n* = 2891, 51.5%), followed by *blaOXA-9* (*n* = 969, 17.3%), *blaOXA-48* (*n* = 800, 14.3%), *blaOXA-232* (*n* = 480, 8.6%), *blaOXA-10* (*n* = 158, 2.8%), *blaOXA-181* (*n* = 146, 2.6%), and *blaOXA-2* (*n* = 108, 1.9%) (Fig. 1A). Variants including *blaOXA-244* (*n* = 12, 0.2%), *blaOXA-245* (*n* = 11, 0.2%), *blaOXA-162* (*n* = 5, 0.1%), *blaOXA-4* (*n* = 5, 0.1%), *blaOXA-926* (*n* = 4, 0.1%), *blaOXA-204* (*n* = 3, 0.1%), *blaOXA-534* (*n* = 3, 0.1%), *blaOXA-58* (*n* = 2, 0.03%), and *blaOXA-320* (*n* = 2, 0.03%) were few. Whereas, the following variant was only detected in single strain which were *blaOXA-21*, *blaOXA-392*, *blaOXA-517*, *blaOXA-519*, *blaOXA-663*, *blaOXA-796*, *blaOXA-827*, *blaOXA-427*, *blaOXA-544*, *blaOXA-436* and *blaOXA-17*.

### Phylogenetic relationship of *blaOXA* variants among global *K. pneumoniae*

**Fig. 2:**
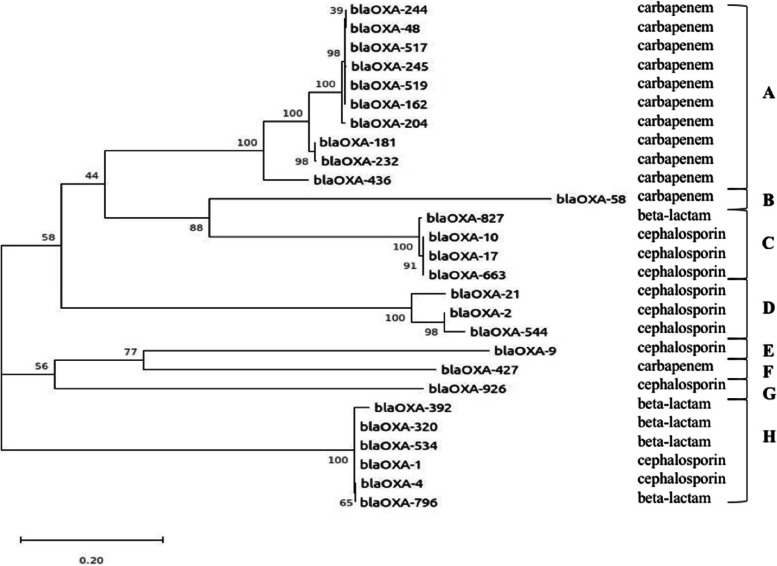
Phylogenetic tree based on SNP sequence of global *blaOXA* variants

The phylogenetic tree showed that *blaOXA* variants could be divided into 8 clades (Fig. 2). Among them, clade A was composed of 10 variants and all of them belonged to carbapenem-hydrolyzing class D ß-lactamase (Group III, *blaOXA-48-like* subgroup). Clade B belonged to Group IV (*blaOXA-51-like* subgroup). Clade C belonged to Group II with the activity of acquired extended spectrum class D ß-lactamases, amongst them, *blaOXA-10-type* oxacillinase, with weak carbapenemases being previously found [17]. Natural variant of *blaOXA-10* was reported to own increased carbapenemase activity and high-level expression of *blaOXA-10* and *blaOXA-10* derivative *blaOXA-663* can confer carbapenem resistance when expressed at sufficiently high levels in the OmpK36 deficiency background [18], indicating that clade C may own the potential to develop to be group III. In clade D, *blaOXA-544* derived from *blaOXA-2* family, as well as *blaOXA-21* all belonged to Group 1, acquired narrow-spectrum oxacillinase without significant activity toward cephalosporins or the carbapenems [19]. The *blaOXA* variants within Clade H may belong to Group I considering the activity of *blaOXA-1* and *blaOXA-4*, although the hydrolyzing activity of *blaOXA-534*, *blaOXA-329*, *blaOXA-320* and *blaOXA-796* remained unknown. Clade E, F and G were composed of single *blaOXA* variant, where, *blaOXA-9* and *blaOXA-926* were narrow-spectrum oxacillinase [20], *blaOXA-427* was a new plasmid-borne carbapenem-hydrolysing class D β-lactamase in *Enterobacteriaceae* [21]. Overall, a relatively independent evolutionary relationship between carbapenem-hydrolyzing oxacillinase *blaOXA* and non-carbapenem-hydrolyzing oxacillinase *blaOXA* were observed. However, several *blaOXA* within Group I and II may develop carbapenem-hydrolyzing activity under the selective pressure of antimicrobial agents.

### The sequence types of *blaOXA*-carrying *K. pneumoniae*

A total of 300 different STs were identified among the 4386 *blaOXA*-carrying *K. pneumoniae* strains. The predominant type was ST11 (*n* = 477, 10.9%), followed by ST258 (*n* = 410, 9.4%), ST15 (*n* = 367, 8.4%), ST307 (*n* = 300, 6.8%), ST147 (*n* = 292, 6.7%), ST16 (*n* = 266, 6.1%) and other STs (Figure S1). In addition, 94 strains were novel or unknown STs (Table S1).

For strains producing different *blaOXA* variants, 2845 *blaOXA-1*-carrying *K. pneumoniae* strains were assigned into 209 STs. Amongst them, ST11 (*n* = 372, 13.1%) was the predominant one, followed by ST307 (*n* = 287,10.2%) (Fig. 1B). Meanwhile, 957 *blaOXA-9*-carrying *K. pneumoniae* strains belonged to 74 STs, with ST258 being accounting for the majority (367/957 38.4%), followed by ST16 (126/957 13.2%) (Fig. 1C). The most extensively studied *blaOXA-48*-carrying *K. pneumoniae* were assigned into 91 STs, with ST11 (136/794, 17.1%) and ST101 (103/794, 13.0%) being the leading STs.

Additionally, the predominant *blaOXA* variant of 477 ST11 strains was *blaOXA-1* (*n* = 378, 79.2%) and the main *blaOXA* variant of 410 ST258 strains was *blaOXA-9* (*n* = 370, 90.2%). The distribution of *blaOXA-1* in ST11 (378/477, 79.2%) was significantly higher than that in ST258 (24/410, 5.9%), the difference was statistically significant (*p* = 0.000). Meanwhile, the prevalence of *blaOXA-9* in ST258 (370/410, 90.2%) was obviously higher than that in ST11 (48/477, 10.1%), the difference was also statistically significant (*p* = 0.000).

### Distribution characteristics of *blaOXA* among global *K. pneumoniae*

**Fig. 3:**
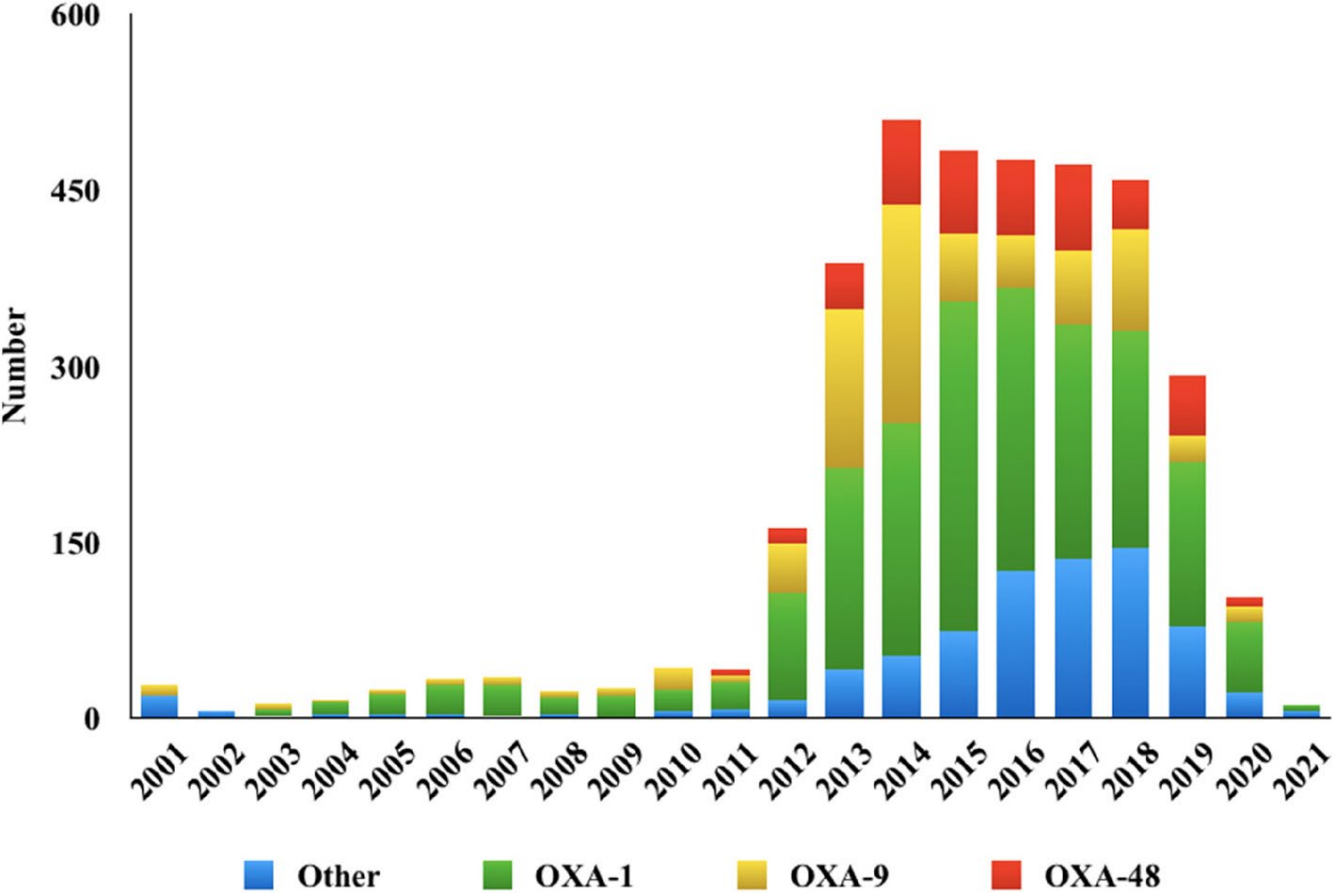
The number of global *K. pneumoniae blaOXA* variants detected from 2001 to 2021

The* blaOXA-9* was the earliest *blaOXA* variants in the genome submitted in USA in 2001 albeit *blaOXA-1* was the first reported one. Since the first identification of *blaOXA-48*-carrying *K. pneumoniae* isolates in 2001, the number of *blaOXA*-carrying *K. pneumoniae* isolates collected from all the specimen types had basically shown an increasing trend till 2014 and had been stable since then (Fig. 3). The predominant *blaOXA-1*, *blaOXA-9* and *blaOXA-48* variants showed the same trend, which should be of concern.

The main host of *blaOXA*-carrying *K. pneumoniae* isolates was homo sapiens (2696/4386, 61.5%), followed by water-related host (60/4386, 1.4%), animal-related host (45/4386, 1.0%) and plant-related host (20/4386, 0.5%). The isolates of homo sapiens were mainly derived from blood, respiratory, alimentary tract, and urinary tract infections (Table 1).

**Table 1:** Source distribution and ratio of global *blaOXA*-carrying *K. pneumoniae* strains in homo sapiens

Specimen type	Strains Number	Ratio (%)
Blood	572	21.2
Urine	464	17.2
Sputum	204	7.6
Rectum	184	6.8
Faeces	136	5.0
Endotracheal aspirate	87	3.2
Wound	69	2.6
Ascitic fluid	36	1.3
Pus	28	1.0
Cerebrospinal fluid	10	0.4
Blank	763	28.3
Others	143	5.4
Total	2696	100

**Fig. 4:**
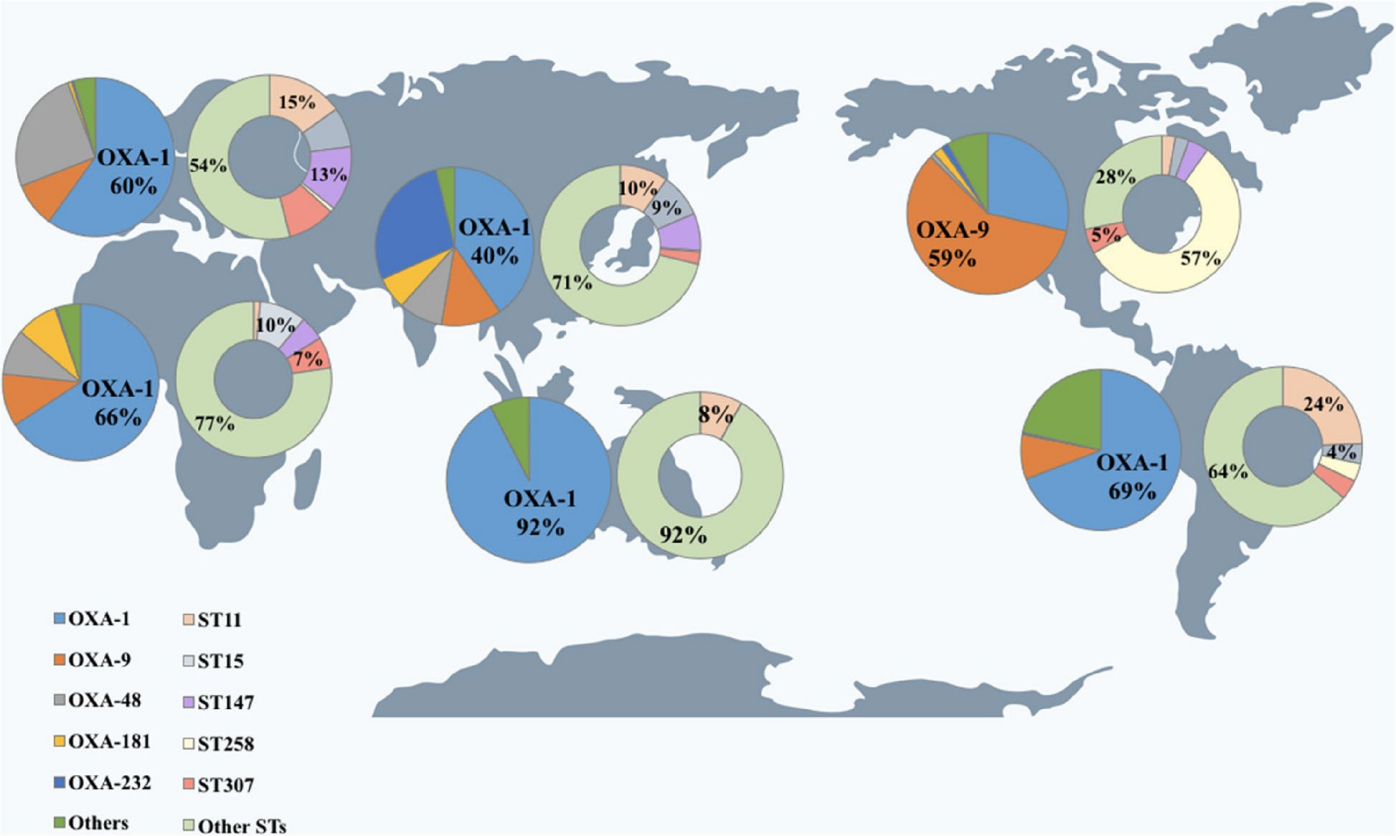
Global distribution of *blaOXA* variants and sequence types of *blaOXA*-carrying *K. pneumoniae* strains

The most predominant *blaOXA-1*-carrying *K. pneumoniae* strains were distributed widely around the world except Northern America, which was relatively rare. The *blaOXA-9*-carrying *K. pneumoniae* strains were mostly found in Northern America rather than other regions, especially, ST258 was mainly distributed in this area. The most studied *blaOXA-48*-carrying *K. pneumoniae* strains were mainly distributed in Europe and Asia. The* blaOXA-232*-carrying *K. pneumoniae* strains and *blaOXA-181*-carrying *K. pneumoniae* strains were almost found in Asian countries (Fig. 4).

### The distribution of antimicrobial resistance determinants among global *blaOXA*-carrying *K. pneumoniae*

Among the 4386 *blaOXA*-carrying *K. pneumoniae* strains, *blaKPC* (*n* = 985, 22.5%) and *blaNDM* (*n* = 732, 16.7%) were the main *bla*CHßLs with *blaVIM* (*n* = 135, 3.1%) and *blaIMP* (*n* = 28, 0.6%) also being identified. Although *aph* (*n* = 5645, 128.7%), *aad* (*n* = 2927, 66.7%) and *aac* (*n* = 2742, 62.5%) were the predominant aminoglycoside resistance genes, *rmt* (*n* = 347, 7.9%), *armA* (*n* = 332, 7.6%), and *ant (2’)-Ia* (*n* = 99, 2.3%) were also found. *qnr* (*n* = 2340, 53.4%), *oqxAB* (*n* = 4131, 94.2%), *aac(6’)-Ib-cr* (*n* = 49, 1.1%) and *qepA* (*n* = 12, 0.3%) were the fluoroquinolone resistance genes.

**Fig. 5:**
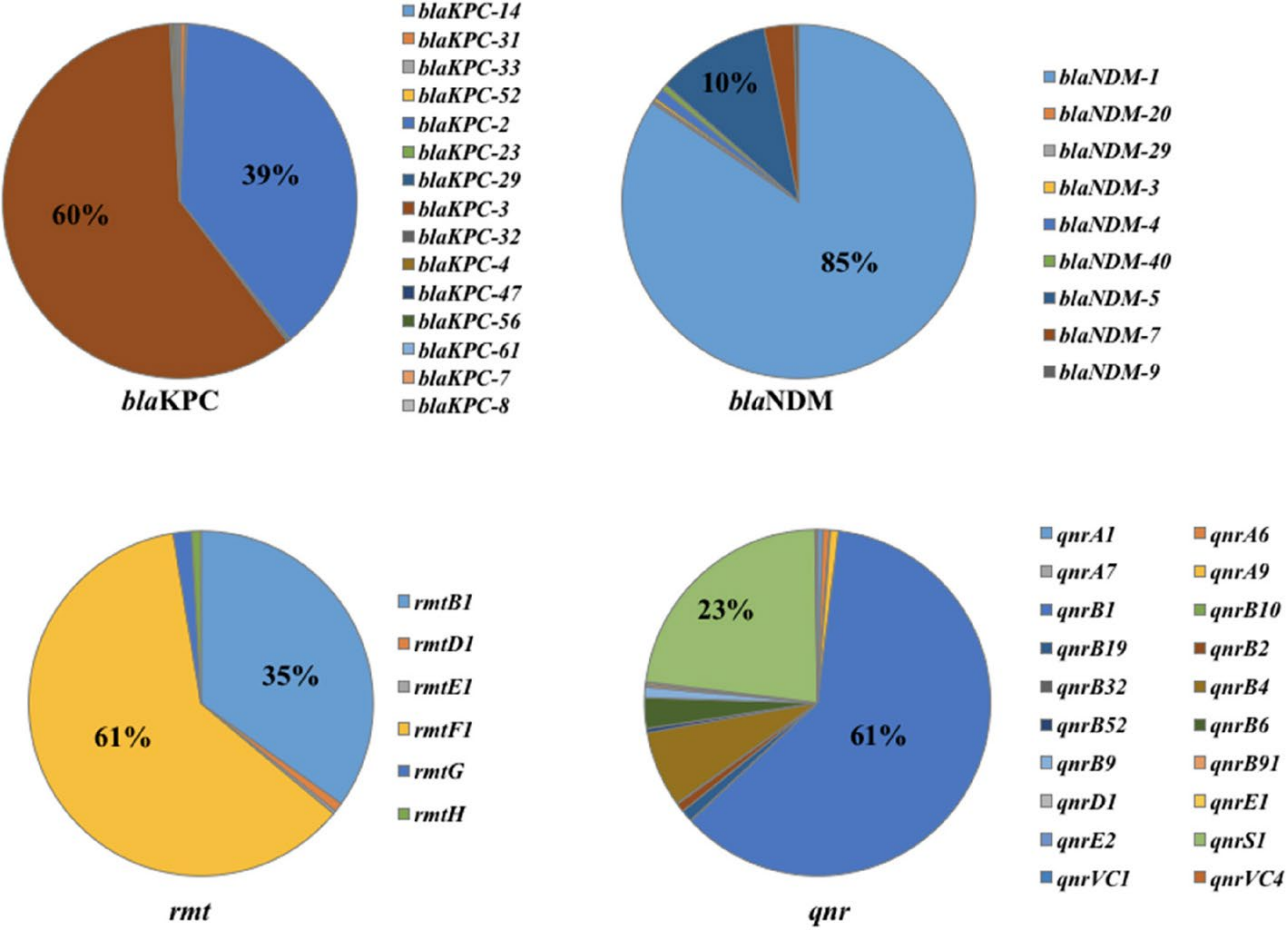
Subtypes of antimicrobial resistance genes among global *blaOXA*-carrying *K. pneumoniae* strains

In detail, the *blaNDM-1* (*n* = 618, 84.1%) and *blaKPC-3* (*n* = 586, 59.6%) were the most common subtypes of *blaKPC* and *blaNDM* respectively. Moreover, *rmtF1* and *rmtB1* were the main variants of *rmt* gene. *qnrB1* and *qnrS1* were the major subtypes of *qnr* gene (Fig. 5).

## Discussion

Current evidence suggests that compared to other Gram-negative opportunists, *K. pneumoniae* has a wider ecological distribution, significantly greater antimicrobial resistance gene diversity and a higher plasmid burden [22]. In recent years, with the carbapenem being frequently used in clinical treatment. Carbapenem-hydrolyzing oxacillinase has been increasingly found, with the outbreaks of infection being reported in Mediterranean and China [2, 5, 6]. As we know that carbapenem-hydrolyzing oxacillinase was commonly prevalent in *Enterobacterale*, especially in *K. pneumoniae* [22]. So the analysis of the distribution of *blaOXA* among the global *K. pneumoniae* based on the genomes could help us to fully illustrate the characteristics of *blaOXA*-carrying *K. pneumoniae*, which is quite useful for formulation and implementation of infection control measures.

In this study, we found that there was a rapid increase in global *blaOXA*-carrying *K. pneumoniae* strains since the first *blaOXA* was identified [23]. Overall, *blaOXA-1* variants were widely distributed around the world. The *blaOXA-9* variants were mostly prevalent in Northern America. Whereas, *blaOXA-48* variants were mainly reported in Europe and Asia which was in accordance with previous studies [5, 6]. Notably, as the *bla*CHßLs, albeit *blaOXA-48*, *blaOXA-232* and *blaOXA-181* were not the most prevalent ones in our study, the emergence of them in different parts of the world has been reported and such genes were found to be most likely underreported due to problems with the laboratory detection of these enzymes [10]. Moreover, we found an increasing trend and wider distribution of the *blaOXA-48* CHßLs, which is consistent with the previous reports [13], indicating an escalating threat of CRKp.

The phylogenetic tree showed a diversity of *blaOXA* variants identified within *K. pneumonae*. Functionally, all the *blaOXA*-variants could be divided into carbapenem-hydrolyzing oxacillinase and non-carbapenem-hydrolyzing one. Within the 29 *blaOXA* variants, 12 out of them have been identified to be *bla*CHßLs, albeit the detailed hydrolyzing activities of 5 variants remained unknown, the increasing diversity and activity towards carbapenem of these *blaOXA*-variants remind us of the continual change trend under the selective pressure of antimicrobial agents.

Diverse STs identified in our study indicated genetic diversity for *blaOXA*-carrying *K. pneumonaie* and a broad host for *blaOXA* variants. Of great concern, high-risk epidemic clones ST11 and ST258 were the predominant STs in our study which have also been the most common host for *bla*CHßLs [24]. As we know that the prevalence of ST11 is high in the continent of Europe, in Greece and Spain, as well as in China [2, 24], and the ST11 clone was reported not only to be the dominant host for *blaKPC* and *blaNDM* [25, 26], but also associated with *blaOXA-48-like* genes (e.g., *blaOXA-163*, *blaOXA-181*) [27, 28]. Although *blaOXA-1* was the main variant among ST11 clones, the frequent occurrence of *blaOXA-48* within global high-risk clone ST11 in our study indicates us that there may be a consistent distribution of *blaOXA*-*bla*CHßLs within *K. pneumonaie.* Additionally, albeit multiple *blaOXA* variants were detected in the ST11 strains, a simultaneous occurrence of *blaOXA-48* and *blaOXA-1* was frequently found, further suggesting the compatibility between these 2 variants within ST11 clone. It has been well known that ST258 clones was the predominant host for *blaKPC* in European and American countries [24], Fortunately, *blaOXA-9* was the main variant within ST258 clone in our study, without other *blaOXA* variants concurrence, which was consistent with the previous study [29, 30], probably indicating a better fitness between *blaOXA-9* and ST258 clones.

Of great concern, more than half of the *blaOXA*-carrying *K. pneumonaie* carried *bla*CHßLs. The frequent occurrence of *blaNDM-1* was mainly among *blaOXA-1* producing *K. pneumonaie*, and high prevalence of *blaKPC-3* was predominantly within *blaOXA-1* and *blaOXA-9* carrying one. which may indicate a better fitness between *blaOXA-1* and *blaNDM-1*, and an association between *blaOXA-1*/ *blaOXA-9* with *blaKPC-3*. Moreover, almost all the *blaOXA*-carrying *K. pneumonaie* carried at least a fluoroquinolone resistance gene, especially *oqxAB*, suggesting that these genes may play an important role in the fluoroquinolone resistance of *K. pneumonaie*. Additionally, albeit *rmt* and *armA* were not the mainly prevalent aminoglycoside resistance gene, such genes frequently should attract our attention based on the conferred resistance to clinically important amikacin.

There are several limitations. First, part of strain information uploaded to GenBank was incomplete, this may result in statistical bias. Second, some *blaOXA*-variants within *K. pneumoniae* have never been reported and the detailed activity remained unknown, since we could just get the genome without the exact strain, so it is hard for us to further analyze the hydrolyzing activity.

In summary, *blaOXA-1* and *blaOXA-9* were the main *blaOXA* among global *K. pneumoniae* with ST11 and ST258 being the predominant STs. *blaOXA* evolved rapidly towards *bla*CHßLs under the selective pressure of antimicrobial agents with *blaOXA-48* and *blaOXA-232* group being increasingly found.

## Supplementary Information

**Additional file 1: FigureS1.** The main sequence types of *blaOXA*-carrying*K. pneumoniae*.

**Additional file 2:** **TableS1.** The sequence types of *blaOXA*-carrying *K. pneumoniae.*

**Additional file 3:** **TableS2.** The accession number and the web link to datasets for the provided name ofall the strains in this study.

## Data Availability

The datasets used and/or analyzed during the current study are available from GenBank and the accession number and web link to datasets for the provided name of these strains were shown in table S2.xlsx.

## Abbreviations

CRE: Carbapenem-resistant Enterobacteriaceae
ESBLs: Extended-spectrum β-lactamases
CHβLs: Carbapenem-hydrolyzing β-lactamase
KPC: *Klebsiella pneumoniae* Carbapenemase
NDM: New Delhi metallo-beta-lactamase
ESBLs: Extended-spectrum β-lactamases
MLST: Multi-locus sequence typing
STs: Sequence types
ARGs: Antibiotics resistance genes
