# Supplementary figures and images for: The distribution characteristics of global blaOXA-carrying Klebsiella pneumoniae
Source: BMC Infect Dis. 2023 Mar 29;23:182. doi: 10.1186/s12879-023-08156-5 (PMC10053090; doi:10.1186/s12879-023-08156-5)

Figure S1. The main sequence types of *blaOXA*-carrying *K. pneumoniae*

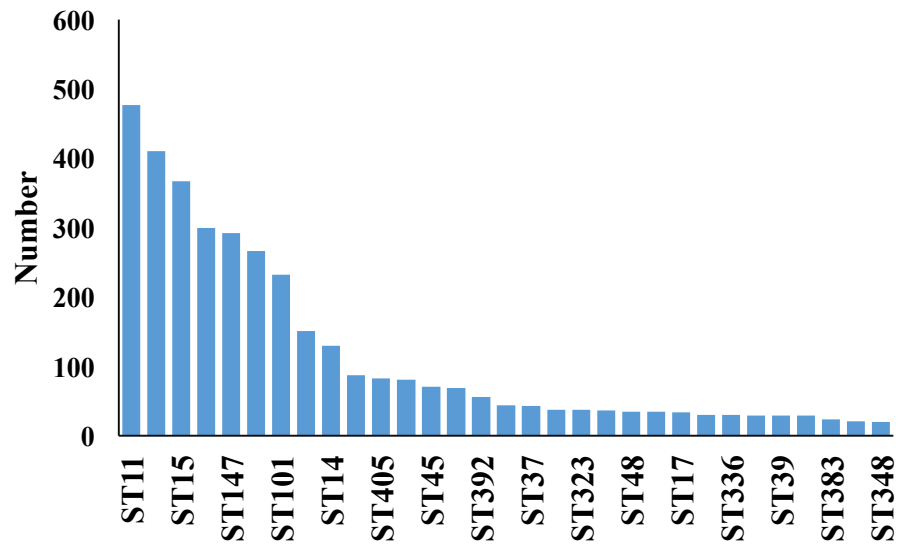

Supplement: Supplementary file 1 — Additional file 1: FigureS1. The main sequence types of blaOXA-carryingK. pneumoniae. [file 12879_2023_8156_MOESM1_ESM.pdf]
