# Supplementary material for: The distribution characteristics of global blaOXA-carrying Klebsiella pneumoniae
Source: BMC Infect Dis. 2023 Mar 29;23:182. doi: 10.1186/s12879-023-08156-5 (PMC10053090; doi:10.1186/s12879-023-08156-5)
Supplement: Supplementary file 2 — Additional file 2: TableS1. The sequence types of blaOXA-carrying K. pneumoniae. [file 12879_2023_8156_MOESM2_ESM.pdf]

Table S1. The sequence types of *blaOXA*-carrying *K. pneumoniae*

ST type	Number
ST11	477
ST258	410
ST15	367
ST307	300
ST147	292
ST16	266
ST101	232
ST231	151
ST14	130
ST395	88
Unknown	86
ST405	83
ST437	81
ST45	71
ST340	69
ST392	56
ST29	44
ST37	43
ST23	38
ST323	38
ST152	37
ST13	35
ST48	35
ST17	34
ST326	30
ST336	30
ST268	29
ST35	29
ST39	29
ST383	24
ST874	21
ST348	20
ST985	18
ST2096	17
ST25	16
ST1427	15
ST1436	15
ST1	14
ST377	14
ST273	13

ST471	13
ST3697	12
ST76	12
ST274	11
ST280	11
ST70	11
ST20	9
ST628	9
Novel	8
ST193	8
ST218	8
ST3805	8
ST391	8
ST104	7
ST290	7
ST34	7
ST711	7
ST86	7
ST111	6
ST1552	6
ST198	6
ST299	6
ST43	6
ST551	6
ST107	5
ST12	5
ST22	5
ST36	5
ST397	5
ST420	5
ST512	5
ST661	5
ST1161	4
ST1626	4
ST1731	4
ST219	4
ST309	4
ST327	4
ST353	4
ST38	4
ST525	4
ST530	4
ST656	4

ST716	4
ST788	4
ST846	4
ST870	4
ST902	4
ST1236	3
ST133	3
ST1426	3
ST1799	3
ST2017	3
ST215	3
ST234	3
ST294	3
ST3650	3
ST394	3
ST427	3
ST429	3
ST485	3
ST5	3
ST560	3
ST831	3
ST105	2
ST1193	2
ST1207	2
ST129	2
ST1307	2
ST163	2
ST1634	2
ST1787	2
ST187	2
ST199	2
ST2167	2
ST221	2
ST2217	2
ST2442	2
ST252	2
ST2719	2
ST277	2
ST2975	2
ST3248	2
ST3410	2
ST3493	2
ST3559	2

ST359	2
ST3721	2
ST3995	2
ST42	2
ST44	2
ST442	2
ST461	2
ST54	2
ST607	2
ST617	2
ST643	2
ST65	2
ST704	2
ST727	2
ST915	2
ST941	2
ST983	2
ST987	2
ST1015	1
ST1047	1
ST1076	1
ST1079	1
ST108	1
ST110	1
ST1199	1
ST1224	1
ST1272	1
ST1303	1
ST1310	1
ST1393	1
ST1399	1
ST1440	1
ST1454	1
ST151	1
ST1518	1
ST1540	1
ST1545	1
ST1564	1
ST158	1
ST1583	1
ST1593	1
ST1709	1
ST1788	1

ST1824	1
ST1836	1
ST1842	1
ST1844	1
ST1855	1
ST188	1
ST1890	1
ST194	1
ST1942	1
ST2118	1
ST2279	1
ST228	1
ST2316	1
ST2333	1
ST2357	1
ST2390	1
ST242	1
ST2426	1
ST244	1
ST253	1
ST2599	1
ST26	1
ST2646	1
ST2695	1
ST27	1
ST2724	1
ST2728	1
ST2739	1
ST281	1
ST2816	1
ST292	1
ST2923	1
ST2948	1
ST295	1
ST3189	1
ST322	1
ST3299	1
ST3335	1
ST3360	1
ST3366	1
ST3385	1
ST3392	1
ST3393	1

ST3395	1
ST347	1
ST3483	1
ST3499	1
ST3576	1
ST3577	1
ST3589	1
ST3592	1
ST3593	1
ST3596	1
ST3600	1
ST3603	1
ST3609	1
ST3623	1
ST3627	1
ST3638	1
ST3668	1
ST3672	1
ST3681	1
ST3691	1
ST3704	1
ST3712	1
ST3713	1
ST3720	1
ST3739	1
ST3740	1
ST3741	1
ST3742	1
ST3751	1
ST3755	1
ST379	1
ST3795	1
ST3796	1
ST3810	1
ST3812	1
ST3830	1
ST3843	1
ST3844	1
ST3847	1
ST40	1
ST4080	1
ST410	1
ST412	1

ST416	1
ST4239	1
ST432	1
ST441	1
ST4422	1
ST443	1
ST4452	1
ST449	1
ST460	1
ST462	1
ST464	1
ST466	1
ST469	1
ST4847	1
ST4873	1
ST490	1
ST500	1
ST514	1
ST515	1
ST5206	1
ST534	1
ST540	1
ST5446	1
ST566	1
ST567	1
ST580	1
ST5822	1
ST592	1
ST624	1
ST629	1
ST659	1
ST726	1
ST73	1
ST77	1
ST789	1
ST791	1
ST8	1
ST833	1
ST834	1
ST855	1
ST857	1
ST881	1
ST882	1

ST893	1
ST896	1
ST920	1
ST995	1
Total	4386
